# Flavonoids Synergistically Enhance the Anti-Glioblastoma Effects of Chemotherapeutic Drugs

**DOI:** 10.3390/biom11121841

**Published:** 2021-12-07

**Authors:** Kevin Zhai, Alena Mazurakova, Lenka Koklesova, Peter Kubatka, Dietrich Büsselberg

**Affiliations:** 1 Department of Physiology and Biophysics, Weill Cornell Medicine-Qatar, Education City, Qatar Foundation, Doha P.O. Box 24144, Qatar; kez4003@qatar-med.cornell.edu; 2 Clinic of Obstetrics and Gynecology, Jessenius Faculty of Medicine, Comenius University in Bratislava, 036 01 Martin, Slovakia; liskova80@uniba.sk (A.M.); koklesova5@uniba.sk (L.K.); 3 Department of Medical Biology, Jessenius Faculty of Medicine, Comenius University in Bratislava, 036 01 Martin, Slovakia; peter.kubatka@uniba.sk

**Keywords:** glioblastoma, glioma, brain cancer, flavonoids, synergy

## Abstract

Flavonoids are polyphenolic plant secondary metabolites with pleiotropic biological properties, including anti-cancer activities. These natural compounds have potential utility in glioblastoma (GBM), a malignant central nervous system tumor derived from astrocytes. Conventional GBM treatment modalities such as chemotherapy, radiation therapy, and surgical tumor resection are beneficial but limited by extensive tumor invasion and drug/radiation resistance. Therefore, dietary flavonoids—with demonstrated anti-GBM properties in preclinical research—are potential alternative therapies. This review explores the synergistic enhancement of the anti-GBM effects of conventional chemotherapeutic drugs by flavonoids. Primary studies published between 2011 and 2021 on flavonoid–chemotherapeutic synergy in GBM were obtained from PubMed. These studies demonstrate that flavonoids such as chrysin, epigallocatechin-3-gallate (EGCG), formononetin, hispidulin, icariin, quercetin, rutin, and silibinin synergistically enhance the effects of canonical chemotherapeutics. These beneficial effects are mediated by the modulation of intracellular signaling mechanisms related to apoptosis, proliferation, autophagy, motility, and chemoresistance. In this light, flavonoids hold promise in improving current therapeutic strategies and ultimately overcoming GBM drug resistance. However, despite positive preclinical results, further investigations are necessary before the commencement of clinical trials. Key considerations include the bioavailability, blood–brain barrier (BBB) permeability, and safety of flavonoids; optimal dosages of flavonoids and chemotherapeutics; drug delivery platforms; and the potential for adverse interactions.

## 1. The Challenges of GBM Therapy and the Potential of Flavonoids

Glioblastoma (GBM) is an astrocyte-derived solid tumor of the brain or spinal cord that occurs at an overall rate of 3.19 cases per 100,000 individuals in the United States. Its incidence varies notably between subpopulations, with males and older individuals at higher risk [1]. GBM is fatal, with median survival times under one year [2].

Currently, conventional medical and surgical interventions predominate in GBM therapy. Standard treatment regimens include (1) radiation therapy with concurrent temozolomide (TMZ) chemotherapy and (2) surgical tumor resection with radiation therapy [3,4]. Recent advances in these therapies have improved patient outcomes; the addition of TMZ, an alkylating agent, to standard radiation-only regimens after 2005 greatly increased survival rates [2]. Nevertheless, conventional interventions remain constrained by GBM’s malignant properties. Surgical methods, for instance, are hindered by widespread tumor invasion and metastasis, while drug and radiation resistance—particularly associated with glioma stem cells (GSCs)—pose challenges for chemo- and radiotherapy [5,6]. Intra- and intertumoral heterogeneity further complicates anti-GBM regimens [6]. Therefore, a need exists for alternative and supportive therapies with the potential to overcome these challenges.

Dietary natural compounds constitute promising candidates in this regard; they have wide-ranging biological properties, including anti-cancer effects [7,8,9,10,11]. Among these compounds, flavonoids—polyphenolic plant secondary metabolites—are of interest. Flavonoids exert anti-cancer effects through chemosensitization, metabolic modulation, metastatic inhibition, and apoptotic induction [12,13]. Based on these well-evidenced oncostatic activities, flavonoids have great potential in modulating GBM cell responses to anti-cancer drugs by overcoming their therapeutic resistance. The efficacy of flavonoids in GBM is well documented in preclinical research [14]. This review aims to complement previous research by focusing on the synergistic efficacy of flavonoids and conventional chemotherapeutics in GBM therapy.

## 2. Study Methodology

Primary studies on flavonoid-chemotherapeutic synergy in GBM were obtained through a PubMed search with the keywords “flavonoid”, “chemo *”, “synerg *” and “glioblastoma” or “glioma.” Approximately 15 articles published from 2011 to 2021 were included. Studies demonstrating the effects of flavonoids alone on GBM—without trials with chemotherapeutic drugs—were excluded.

## 3. Flavonoids and Chemotherapeutics in GBM Therapy

### 3.1. Flavonoids

**Figure 1:**
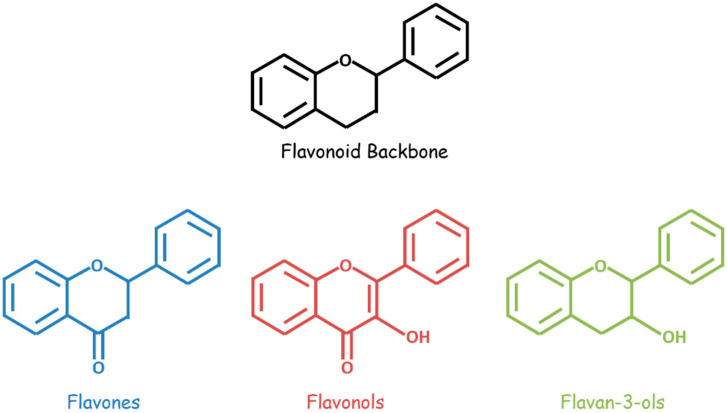
General structures of flavonoids (black), flavones (blue), flavonols (red), and flavan-3-ols (green).

Bioactive flavonoids occur in fruits, vegetables, and other natural plant products and are unified by a three-ring structural backbone that includes two phenyl rings and one central heterocyclic ring. These compounds are classified based on structural differences—related primarily to the presence and positioning of substituents on the heterocycle (Figure 1). A variety of flavonoids, including flavan-3-ols, flavones, isoflavones, flavonols, flavonol glycosides, and flavonolignans, demonstrate anti-GBM effects combined with chemotherapeutic drugs in vitro and/or in vivo (Table 1).

Flavan-3-ols comprise a class of flavonoids with a hydroxyl substituent at the third position of the heterocyclic ring. One flavan-3-ol of particular interest in GBM therapy, epigallocatechin-3-gallate (EGCG), occurs predominantly in green tea and exerts pro-apoptotic, antiproliferative, and antioxidant effects in cancerous cells [15].

In contrast, the class of flavones and isoflavones includes flavonoid compounds with a ketone substituent at the fourth position of the heterocycle. Two flavones and one isoflavone are of interest in synergistic GBM therapy. Chrysin, a flavone found in passionflower, honey, and propolis, has anti-cancer, neuroprotective, and other beneficial properties [16]. Similarly, hispidulin, a flavone from *Grindelia*, *Artemisia*, and *Salvia* plants, exerts anti-cancer, antifungal, antioxidant, and anti-inflammatory effects; it is moreover a benzodiazepine (BZD) receptor ligand [17]. Finally, formononetin, an O-methylated isoflavone, and phytoestrogen in legumes and clovers, have anti-cancer properties [18].

Flavonols have both the third position hydroxyl substituent of flavan-3-ols and the fourth position ketone substituent of flavones. Flavonols and flavonol glycosides, including quercetin, rutin, and icariin, are of interest in synergistic GBM therapy. Quercetin, a flavonol found in oak, berries, apples, grapes, cilantro, and onions, exerts antioxidant, antihistamine, anti-inflammatory, and anti-cancer activities [19]. Rutin, the glycoside of quercetin, has similar biological activities and occurs in rue, apples, buckwheat, and citrus fruits [21]. Another flavonol glycoside, icariin, is commonly found in horny goat weed; in addition to its anti-cancer properties, it has aphrodisiac, neuroprotective, and anti-osteoporotic effects [20].

Finally, flavonolignans are flavonoid derivatives with both flavonoid and phenylpropanoid structural components. Silibinin, a flavonolignan of interest in synergistic GBM therapy, is found in milk thistle seeds and has broad anti-cancer and antimetastatic effects [22].

### 3.2. Chemotherapeutics

**Figure 2:**
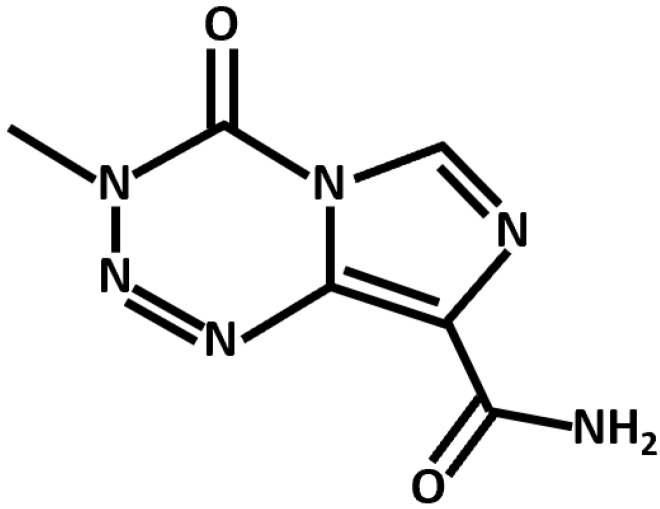
Chemical structure of TMZ, an alkylating agent and anti-GBM chemotherapeutic.

Conventional chemotherapeutics leverage diverse mechanistic pathways to exert their anti-cancer effects. TMZ, the canonical anti-GBM drug, is an alkylating agent that induces apoptotic cell death through the p53-dependent and *O*6-methylguanine-based activation of the Fas/caspase 8 pathway (Figure 2) [23]. In addition, several noncanonical and repurposed drugs hold promise in synergistic GBM therapy (Table 2).

One such drug, arsenic trioxide (ATO), exerts pleiotropic anti-cancer effects through ROS generation and cell cycle regulation [24]. In glioma cells, ATO induces caspase-independent autophagic cell death [29]. Moreover, combinations of ATO and TMZ and ATO and vismodegib exert synergistic effects against GBM growth in vivo [30].

Chloroquine, another compound of interest, is a repurposed antimalarial drug that induces p53-dependent apoptosis and disrupts the mitochondrial membrane potential in glioma cells [25]. In conjunction with standard radiation and chemotherapeutic treatment regimen, a recent clinical trial examined its efficacy against GBM [31].

As a widely utilized chemotherapeutic, cisplatin—a platinum-based DNA alkylating agent—was clinically trialed in various cancers, including GBM. Mechanistically, cisplatin’s anti-GBM effects arise from p53-dependent apoptosis [26].

Similarly, the naturally derived topoisomerase II inhibitor etoposide was extensively clinically trialed in GBM. Etoposide induces glioma cell apoptosis through sequential ceramide formation, Bax/Bcl-2 modulation, cytochrome c release, and caspase activation [27].

Finally, sodium butyrate (NaB) is a short-chain fatty acid histone deacetylase inhibitor that reduces glioma cell proliferation, migration, and cell cycle progression [28]. While NaB has anti-GBM potential, its effects remain unsubstantiated by clinical trials at this time.

## 4. Mechanisms of GBM and Synergistic Flavonoid-Chemotherapeutic Effects

### 4.1. Mechanisms of GBM

**Figure 3:**
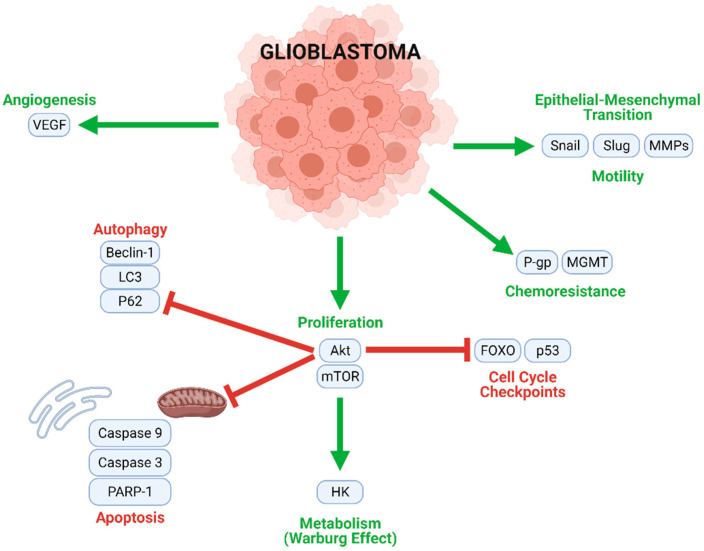
Multiple intracellular processes contribute to GBM tumorigenesis and progression. Mechanisms contributing to proliferation, chemoresistance, metabolism, angiogenesis, and motility (migration) are upregulated in GBM cells, while cell cycle checkpoints, autophagy, and apoptosis are inhibited.

GBM tumorigenesis, progression, and metastasis are driven by numerous interconnected signaling mechanisms (Figure 3). Rapid cell proliferation, an essential process at all stages of GBM development, is mediated by the Akt/mammalian target of rapamycin (mTOR), nuclear factor κappa of activated B cells (NF-κB), and other similar pathways. Uncontrolled proliferation of this nature is enabled by the inhibition of normal cell cycle controls (such as FOXO and p53), and the downregulation of key actors in autophagic (LC3, Beclin-1, and P62) and apoptotic (caspases) cell death. Moreover, a metabolic transition to aerobic glycolysis (the Warburg effect) energetically sustains rapid GBM cell division. Angiogenic and neovascular processes—stimulated mainly by vascular endothelial growth factor (VEGF) signaling—ensure oxygen and nutrient transport to growing tumors. GBM cells may further develop chemoresistance; this often occurs through *O*6-methylguanine methyltransferase (MGMT), which confers resistance to alkylating agents and/or P-glycoprotein (P-gp), which enhances drug efflux from the cells. Finally, Snail, Slug, and matrix metalloproteinases (MMPs) contribute to the epithelial–mesenchymal transition (EMT), which causes GBM cells to develop migratory and invasive phenotypes.

### 4.2. Flavonoids and TMZ

**Figure 4:**
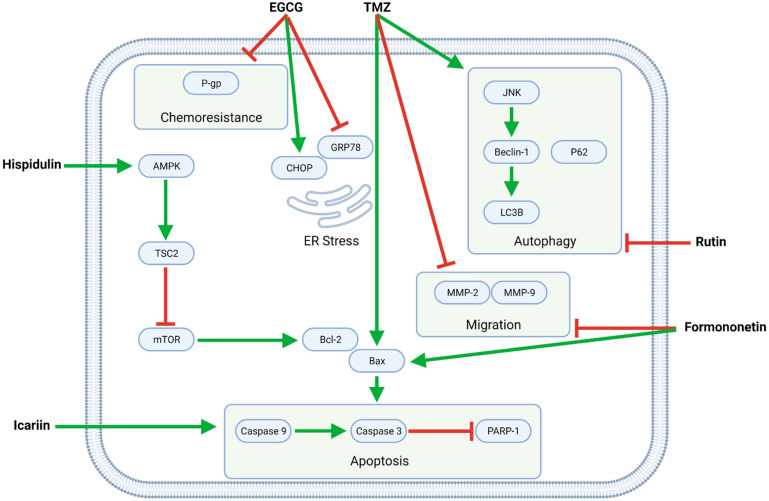
The flavonoids EGCG, formononetin, hispidulin, icariin, and rutin exert pleiotropic anti-GBM effects combined with TMZ. Formononetin, hispidulin, and icariin synergistically enhance TMZ-mediated apoptosis by increasing the Bax/Bcl-2 ratio and activating caspases; formononetin additionally potentiates TMZ’s anti-migratory effects. Moreover, EGCG downregulates P-gp, thereby increasing the sensitivity of (otherwise resistant) GBM cells to TMZ. Finally, rutin inhibits TMZ-induced autophagy and, as such, promotes apoptotic cell death.

Several flavonoids—EGCG, formononetin, hispidulin, icariin, and rutin—synergize with TMZ in modulating intracellular pathways related to proliferation, apoptosis, autophagy, migration, and chemoresistance (Figure 4, Table 3).

#### 4.2.1. TMZ and EGCG

The green tea catechin EGCG potentiates TMZ’s anti-GBM effects by upregulating C/EBP homologous protein (CHOP) and downregulating glucose-regulated protein 78 (GRP78) and consequently inducing endoplasmic reticulum (ER) stress, which contributes to apoptosis [32]. Moreover, EGCG mitigates GBM cell chemoresistance by downregulating P-gp [38]. While EGCG’s chemosensitizing effects were observed in U87 glioma-like stem cells (GLSC), its synergistic pro-apoptotic effects are demonstrable in murine intracranial (orthotopic) U87 and U251 xenograft models and increased the survival times of said tumor-bearing mice [32]. EGCG additionally inhibits MGMT, a regulator of TMZ resistance in GBM, and thus reverses TMZ resistance in MGMT-positive GBM-XD and T98G cells via the Wingless-related integration site (WNT)/β-catenin pathway [42].

#### 4.2.2. TMZ and Formononetin

In C6 cells, formononetin enhances TMZ’s pro-apoptotic and anti-migratory effects by upregulating Bax and cleaved caspases and downregulating MMPs, respectively [39,40]. Similarly, formononetin and calycosin together potently increase the effectiveness of TMZ in C6 cells and a murine C6 xenograft model [40].

#### 4.2.3. TMZ and Hispidulin

Hispidulin potentiates the pro-apoptotic activity of TMZ by upregulating 5′ adenosine monophosphate-activated protein kinase (AMPK), whose downstream effector tuberous sclerosis 2 (TSC2) inhibits mTOR and consequently downregulates the antiapoptotic protein Bcl-2, allowing for an increased Bax/Bcl-2 ratio that is favorable for apoptosis. Furthermore, TMZ and hispidulin induce G2/M phase cell cycle arrest, as demonstrated in SHG44 cells [37].

#### 4.2.4. TMZ and Icariin

While icariin functions primarily as an apoptotic enhancer in conjunction with TMZ, it also inhibits NF-κB-mediated proliferation and reduces migration and invasion in U87MG cells [36].

#### 4.2.5. TMZ and Flavonoid-Rich Extracts

Together, TMZ and Marcela extract (which contains a mixture of flavonoids) increase apoptosis by upregulating cleaved caspases in vitro [34]. Moreover, flavonoid-rich pine needle water extract (PWE) sensitizes GBM8901 cells to TMZ by downregulating autophagy [41].

#### 4.2.6. TMZ and Rutin

TMZ increases both apoptotic and autophagic cell death in GBM cells. At the same time, the flavonoid rutin shifts the balance toward apoptosis by upregulating caspases and inhibiting autophagy by downregulating light chain 3 (LC3) and c-Jun N-terminal kinase (JNK). As such, TMZ and rutin synergistically decrease tumor weight and volume in both intracranial (orthotopic) and subcutaneous (heterotopic) murine xenograft models [33].

#### 4.2.7. TMZ and Silibinin (LN229)

Silibinin enhances TMZ-induced apoptosis by downregulating the apoptotic inhibitor Survivin [35].

### 4.3. Other Combinations of Flavonoids and Chemotherapeutics

Six additional flavonoid–chemotherapeutic combinations with promising synergistic anti-GBM effects are quercetin and chloroquine, quercetin and NaB, *Gardenia jasminoides* (GJ) extract and cisplatin, silibinin and etoposide, silibinin and ATO, and chrysin and ATO (Table 4).

#### 4.3.1. Quercetin and Chloroquine

**Figure 5:**
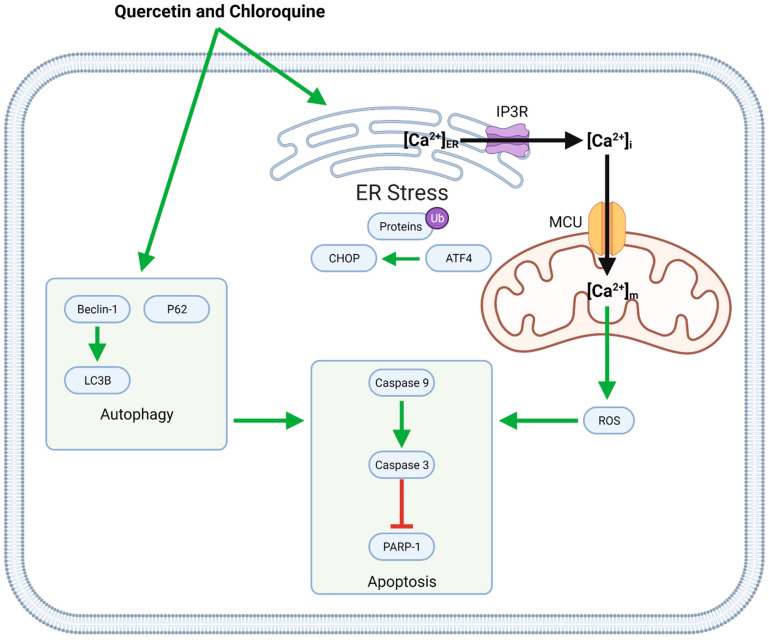
Synergistic anti-GBM effects of quercetin and chloroquine mediated by the induction of ER stress and autophagy. ER stress causes the release of Ca^2+^ into the intracellular space; some of this Ca^2+^ enters mitochondria via the MCU, leading to mitochondrial ROS generation. In this case, both mitochondrial ROS and autophagic mechanisms contribute to apoptotic cell death.

Co-administration of quercetin with chloroquine causes both apoptotic and autophagic cell death (Figure 5). These compounds induce autophagy by upregulating Beclin-1, LC3, and P62 and increasing apoptosis through ER stress and mitochondrial dysfunction. ER stress, associated with the upregulation of ATF4 and CHOP and the buildup of ubiquitinated proteins, leads to calcium (Ca^2+^) release into the cytosol. Intracellular Ca^2+^ then enters mitochondria via the mitochondrial Ca^2+^ uniporter (MCU); increased mitochondrial calcium concentrations ([Ca^2+^]_m_) upregulate the generation of reactive oxygen species (ROS), which in turn contribute to caspase-induced apoptosis [43].

#### 4.3.2. GJ and Cisplatin

Flavonoid-rich GJ extract synergistically enhances cisplatin-induced apoptotic cell death through the upregulation of active caspases. However, GJ–cisplatin synergy differs from quercetin–chloroquine synergy. GJ inhibits cisplatin-induced autophagy in favor of apoptosis in a manner consistent with that of rutin–TMZ synergy (Figure 3) [44].

#### 4.3.3. Quercetin and NaB

Similarly to GJ extract, quercetin synergistically enhances apoptosis by upregulating caspases and downregulating Survivin and Bcl-2, and concurrently inhibits NaB-induced autophagy by downregulating LC3 and Beclin-1 [45]. Cellular senescence is another option for GBM therapy; NaB and quercetin together induce senescence-like growth arrest in U87 and C6 cells [48].

#### 4.3.4. Silibinin and Etoposide; Silibinin and ATO; Chrysin and ATO

Silibinin–etoposide, silibinin–ATO, and chrysin–ATO combinations reduce GBM cell viability in vitro; silibinin and ATO, in particular, induce apoptosis and inhibit cell migration and metabolism [47]. However, the mechanisms of action of silibinin–etoposide and chrystin–ATO combinations remain largely unclarified [35,46].

## 5. Key Considerations and Challenges

While recent preclinical findings on flavonoid–chemotherapeutic synergy in GBM therapy are promising, many mechanistic unknowns, intricacies, and challenges remain. One major limitation of current knowledge is inherent in the literature: all of the reviewed studies are in vitro or in vivo preclinical studies utilizing statistical significance as a threshold for treatment efficacy. However, statistical significance does not necessarily correspond to clinical significance, and laboratory studies are often insufficient to predict outcomes under genuine (and highly variable) physiological conditions.

Another pertinent consideration related to the preclinical literature is the justification of synergistic effects. The data in Table 3 and Table 4 represent synergism as defined in the reviewed primary studies. However, it is worth noting that synergism is poorly defined at present, with limited consensus across the scientific and biomedical communities; this ambiguity leads to the mischaracterization of additive and other combined effects as synergistic effects in some cases. As such, standardized measures of synergism have been proposed. One auspicious measure developed by Chou and Talalay evaluates synergism as a mass action—rather than statistical—phenomenon, using a combination index (CI) rather than *p* values [49]. Notably, a significant proportion of the reviewed studies utilized CI to measure synergism (or lack thereof). Zhang et al. presented CI < 1 for combinations of 40–320 µM formononetin and 250–2000 µM TMZ, indicating synergy between the two compounds [39]. Similarly, Wang et al. demonstrated synergy between hispidulin and TMZ, with CI = 0.584 [37]. Synergistic effects of EGCG–TMZ, quercetin–chloroquine, quercetin–NaB, chrysin–ATO, and silibinin–ATO combinations were likewise justified with CI < 1 [32,43,45,46].

Concerning the flavonoids themselves, their consideration as medicinal agents necessitates evaluating their toxicity, blood–brain barrier (BBB) permeability, bioavailability, and potential adverse effects under said physiological conditions. Most of the flavonoids included in this review are nontoxic: chrysin at up to 400–500 mg per day, EGCG at 338 mg, quercetin at 5000 mg, rutin at 1000 mg, and silibinin at 20 mg/kg [16,50,51,52,53]. Icariin is well tolerated at lower doses; however, gastrointestinal side effects may occur at 1,680 mg [54]. Importantly, formononetin administration poses a risk of allergic immune responses through pro-inflammatory cytokines such as interleukin 4 (IL-4) [55]. Finally, the toxicity profile of hispidulin requires further assessment [56].

Beyond toxicity, the potential physiological side effects of flavonoids—both beneficial and detrimental—merit consideration. Hispidulin, for instance, is a BZD receptor antagonist with anti-convulsive effects in vivo [57]. Another flavonoid, formononetin, is a phytoestrogen. While this flavonoid exerts neuroprotective effects through estrogen receptor βeta (ERβ)-dependent inhibition of NF-κB activity and microglia-induced neuroinflammation, it may also promote angiogenesis and endothelial cell proliferation (both potentially detrimental) via estrogen receptor αlpha (Erα) [58,59].

Nontoxicity and a favorable side effect profile constitute the baseline for human consumption; however, effective anti-GBM agents must have high bioavailability (to be present in sufficient doses following oral administration) and BBB permeability (to enter the brain from the bloodstream). Flavonoids and other natural compounds are significantly limited by their low bioavailability and poor aqueous solubility; the bioavailabilities of chrysin, EGCG, formononetin, hispidulin, icariin, rutin, and silibinin are accordingly poor [16,18,22,52,56,60,61,62]. Extensive metabolism in the intestine, colon, and liver (with the participation of gut microbiota) further limits the bioavailability of these flavonoids [13]. In this regard, a cooperative gut microbiome is essential for their bioavailability and absorption [63]. Quercetin’s bioavailability is comparatively better but remains constrained by intestinal efflux and biliary excretion [64]. More promisingly, EGCG, hispidulin, icariin, quercetin, and rutin can cross the BBB; silibinin cannot, while the permeability of chrysin and formononetin remains unclear [17,65,66,67,68]. In this light, developing novel formulations to enhance the bioavailability and brain delivery of flavonoids is of key interest in advancing synergistic anti-GBM therapy. Current research particularly highlights the potential of nanotechnology approaches to this end [12].

Although flavonoids are associated with some challenges, especially in the clinical sphere, they can confront GBM drug resistance, which hinders current conventional therapies. TMZ’s introduction, for instance, improved therapeutic outcomes; however, TMZ resistance in GBM—mediated by the overexpression of MGMT and alkylpurine-DNA-N glycosylase (APNG), which repair TMZ-induced DNA lesions and thereby prevent apoptosis—is now well documented [69]. Cisplatin resistance via hypoxia-inducible factors 1 and 2 (HIF-1/2) and cluster of differentiation 133 (CD133) is also reported in GBM cell lines [70]. Moreover, an etoposide-resistant glioma cell line has been established [71]. Flavonoids hold promise in overcoming these types of resistance, as they downregulate key factors such as MGMT and P-gp and can therefore serve as chemosensitizers.

Taken together, the criteria of efficacy, nontoxicity, BBB permeability, and bioavailability suggest that (1) rutin and TMZ and (2) EGCG and TMZ are auspicious combinations. Rutin and EGCG are nontoxic, have favorable side effect profiles, and can cross the BBB. However, further preclinical experiments and eventually clinical trials are necessary to substantiate the efficacy and safety of these and other flavonoid–chemotherapeutic combinations.

## 6. Conclusions and Outlook

Despite recent medical advances, GBM’s prognosis remains poor. Extensive tumor invasiveness and therapeutic resistance hinder conventional drug, radiation, and surgical therapies. In this regard, flavonoids hold potential as supportive agents that can mitigate the numerous challenges posed by GBM. The flavonoids chrysin, EGCG, formononetin, hispidulin, icariin, quercetin, rutin, and silibinin demonstrate synergistic anti-GBM effects in conjunction with TMZ, cisplatin, chloroquine, etoposide, NaB, and ATO. These beneficial effects are mediated by the enhancement of apoptosis and the reduction of proliferation, migration, and chemoresistance. As such, flavonoids could enhance individual outcomes of GBM therapy, especially by overcoming therapeutic resistance.

While these findings are promising, supportive evidence for flavonoid–chemotherapeutic synergy is currently limited to the preclinical literature. It is additionally worth noting that although many flavonoids exert anti-GBM effects, only some have been evaluated as potential synergistic agents. As such, forward-looking studies should clarify the synergistic effects of promising yet underinvestigated flavonoids. Furthermore, rigorous evaluation of the physiological properties of flavonoids—including toxicity, side effects, bioavailability, and BB permeability—is necessary on the path toward clinical implementation. If and when appropriate, clinical trials should investigate and confirm the safety and therapeutic efficacy of flavonoid–chemotherapeutic combinations.

## Figures and Tables

**Table 1:** Classes and sources of eight flavonoids that synergize with chemotherapeutics to inhibit GBM.

Flavonoid	Class	Canonical or Common Source	Reference
EGCG	Flavan-3-ol	Green and white tea	[15]
Chrysin	Flavone	Passionflower (*Passiflora)*	[16]
Hispidulin	Flavone	Gumweed (*Grindelia argentina*)	[17]
Formononetin	Isoflavone	Red clover (*Trifolium pratense*)	[18]
Quercetin	Flavonol	Oak (*Quercus*)	[19]
Icariin	Flavonol Glycoside	Horny goat weed (*Epimedium*)	[20]
Rutin	Flavonol Glycoside	Rue (*Ruta graveolens*)	[21]
Silibinin	Flavonolignan	Milk thistle (*Silybum marianum*)	[22]

**Table 2:** Classes and functions of chemotherapeutic drugs that have synergistic anti-GBM potential in combination with flavonoids.

Chemotherapeutic	Class	Primary Function	Reference
ATO	Arsenic compounds	Multimodal	[24]
Chloroquine	Anti-malarials	Autophagy inhibitor	[25]
Cisplatin	Platinum compounds	Alkylating agent	[26]
Etoposide	Natural product derivatives	Topoisomerase II inhibitor	[27]
Sodium Butyrate (NaB)	Short-chain fatty acids	Histone deacetylase inhibitor	[28]
TMZ	Purine analogs	Alkylating agent	[23]

**Table 3:** Mechanistic anti-GBM effects of flavonoid-TMZ combinations, as demonstrated in vitro and in vivo.

Effect	Cell Line	Flavonoid	Flavonoid Conc.	TMZ Conc.	Source
Increases survival time	Intracranial U87 xenografts, nude mice	EGCG	50 mg/kg	5 mg/kg	[32]
	Intracranial U251 xenografts, nude mice	EGCG	50 mg/kg	5 mg/kg	[32]
Decreases tumor volume	Subcutaneous U87 xenografts, BALB/c mice	Rutin	20 mg/kg	55 mg/kg	[33]
Decreases tumor weight	Subcutaneous U87 xenografts, BALB/c mice	Rutin	20 mg/kg	55 mg/kg	[33]
	Intracranial U87 xenografts, BALB/c mice	Rutin	20 mg/kg	55 mg/kg	[33]
Increases cell death/dec viability	C6	Marcela Extract	10, 20, 50 µg/mL	200 µM	[34]
	U87	Marcela Extract	10, 20, 50 µg/mL	200 µM	[34]
	U251	Marcela Extract	50 µg/mL	100 µM	[34]
	U87MG	Rutin	50, 100, 200 µM	63, 250, 500, 1000 µM	[33]
	D54MG	Rutin	50, 100, 200 µM	63, 125, 250, 500, 1000 µM	[33]
	U251MG	Rutin	50, 100, 200 µM	63, 125, 250, 500, 1000 µM	[33]
	LN229	Silibinin	50 µM	10, 25, 50 µM	[35]
	TR-LN229	Silibinin	50 µM	10, 25, 50 µM	[35]
	U87	Silibinin	50 µM	25, 50 µM	[35]
	U87MG	Icariin	10 µM	200 µM	[36]
	SHG44	Hispidulin	40 µM	100 µM	[37]
	U87 GSLC	EGCG	100 µM	100 µM	[38]
	U251	EGCG	10, 20 µM	20, 40 µM	[32]
	C6	Formononetin	40, 80, 160, 320 µM	125, 250, 500, 1000, 2000 µM	[39,40]
	GBM8901	PWE	50 µg/mL	100, 150, 200 µM	[41]
Decreases colony formation	U87	Marcela Extract	10, 20, 50 µg/mL	50 µM	[34]
Decreases proliferation	U87MG	Icariin	10 µM	200 µM	[36]
Increases apoptosis	U87MG	Icariin	10 µM	200 µM	[36]
	SHG44	Hispidulin	40 µM	100 µM	[37]
	U251	EGCG	20 µM	100 µM	[32]
	C6	Formononetin	40, 80 µM	125, 500 µM	[39,40]
Upregulates (c-)caspase 3 (protein)	C6	Marcela Extract	50 µg/mL	200 µM	[34]
	U251	Marcela Extract	50 µg/mL	100 µM	[34]
	U87	Rutin	100, 200 µM	500 µM	[33]
	U87MG	Icariin	10 µM	200 µM	[36]
	C6	Formononetin	40, 80 µM	125, 500 µM	[39,40]
Upregulates (c-)caspase 9 (protein)	C6	Formononetin	40, 80 µM	125, 500 µM	[39,40]
Upregulates (c-)PARP (protein)	U87MG	Icariin	10 µM	200 µM	[36]
Upregulates Bax (protein)	C6	Formononetin	40, 80 µM	125, 500 µM	[39,40]
Downregulates Bcl-2 (protein)	SHG44	Hispidulin	40 µM	100 µM	[37]
	C6	Formononetin	40, 80 µM	125, 500 µM	[39,40]
Downregulates Survivin (protein)	LN229	Silibinin	50 µM	50 µM	[35]
Downregulates LC3-II (protein)	U87	Rutin	100, 200 µM	500 µM	[33]
	GBM8901	PWE	50 µg/mL	100 µM	[41]
Downregulates Beclin-1 (protein)	GBM8901	PWE	50 µg/mL	100 µM	[41]
Downregulates P62 (protein)	GBM8901	PWE	50 µg/mL	100 µM	[41]
Downregulates (p-)JNK (protein)	U87	Rutin	100, 200 µM	500 µM	[33]
Upregulates CHOP (protein)	Intracranial U87 xenografts, nude mice	EGCG	50 mg/kg	5 mg/kg	[32]
	Intracranial U251 xenografts, nude mice	EGCG	50 mg/kg	5 mg/kg	[32]
Downregulates GRP78 (protein)	Intracranial U87 xenografts, nude mice	EGCG	50 mg/kg	5 mg/kg	[32]
	Intracranial U251 xenografts, nude mice	EGCG	50 mg/kg	5 mg/kg	[32]
Upregulates (p-)AMPK (protein)	SHG44	Hispidulin	40 µM	100 µM	[37]
Downregulates (p-)mTOR (protein)	SHG44	Hispidulin	40 µM	100 µM	[37]
Decreases cell migration	U87MG	Icariin	10 µM	200 µM	[36]
	C6	Formononetin	40, 80 µM	125, 500 µM	[39,40]
Downregulates MMP-2 (protein)	C6	Formononetin	40, 80 µM	125, 500 µM	[39,40]
Downregulates MMP-9 (protein)	C6	Formononetin	40, 80 µM	125, 500 µM	[39,40]
Decreases cell invasion	U87MG	Icariin	10 µM	200 µM	[36]
Increases G2/M phase arrest	SHG44	Hispidulin	40 µM	100 µM	[37]
Downregulates NF-κB	U87MG	Icariin	10 µM	200 µM	[36]
Downregulates P-gp	U87 GSLC	EGCG	100 µM	100 µM	[38]

**Table 4:** Synergistic anti-GBM effects of quercetin–chloroquine, quercetin–NaB, GJ–cisplatin, silibinin–etoposide, silibinin–ATO, and chrysin–ATO combinations, as demonstrated in vitro.

Effect	Cell Line	Flavonoid	Flavonoid Conc.	Drug	Drug Conc.	Source
Increases cell death/dec viability	T98G	Quercetin	25, 50, 100 µM	Chloroquine	10, 20, 40 µM	[43]
	U251MG	Quercetin	25, 50, 100 µM	Chloroquine	10, 20, 40 µM	[43]
	U373MG	Quercetin	25, 50, 100 µM	Chloroquine	10, 20, 40 µM	[43]
	U87MG	GJ	500 µg/mL	Cisplatin	1 µM	[44]
	U373MG	GJ	500 µg/mL	Cisplatin	1 µM	[44]
	C6	Quercetin	12.5, 25, 50, 100 µM	NaB	1, 3, 5, 8 mM	[45]
	T98G	Quercetin	12.5, 25, 50, 100 µM	NaB	1, 3, 5, 8 mM	[45]
	A172	Chrysin	2, 5, 10, 20 µM	ATO	1, 5, 10, 20 µM	[46]
	A172	Silibinin	10, 20, 50 µM	ATO	1, 5, 10, 20 µM	[46]
	LN229	Silibinin	50 µM	Etoposide	25, 50 µM	[35]
Increases apoptosis	C6	Quercetin	25 µM	NaB	1 mM	[45]
	T98G	Quercetin	25 µM	NaB	1 mM	[45]
	U87MG	Silibinin	75 µM	ATO	1, 2 µM	[47]
Upregulates (c-)caspase 3 (protein)	C6	Quercetin	25 µM	NaB	1 mM	[45]
	T98G	Quercetin	25 µM	NaB	1 mM	[45]
	U87MG	GJ	500 µg/mL	Cisplatin	1 µM	[44]
	U373MG	GJ	500 µg/mL	Cisplatin	1 µM	[44]
	U87MG	Silibinin	75 µM	ATO	1, 2 µM	[47]
Upregulates (c-)caspase 9 (protein)	U87MG	GJ	500 µg/mL	Cisplatin	1 µM	[44]
	U373MG	GJ	500 µg/mL	Cisplatin	1 µM	[44]
Downregulates PARP (protein)	C6	Quercetin	25 µM	NaB	1 mM	[45]
	T98G	Quercetin	25 µM	NaB	1 mM	[45]
Upregulates (c-)PARP (protein)	U87MG	GJ	500 µg/mL	Cisplatin	1 µM	[44]
	U373MG	GJ	500 µg/mL	Cisplatin	1 µM	[44]
Upregulates Bax (protein)	C6	Quercetin	25 µM	NaB	1 mM	[45]
	T98G	Quercetin	25 µM	NaB	1 mM	[45]
Downregulates Bcl-2 (protein)	C6	Quercetin	25 µM	NaB	1 mM	[45]
	T98G	Quercetin	25 µM	NaB	1 mM	[45]
	U87MG	Silibinin	75 µM	ATO	2 µM	[47]
Downregulates Survivin (protein)	C6	Quercetin	25 µM	NaB	1 mM	[45]
	T98G	Quercetin	25 µM	NaB	1 mM	[45]
	U87MG	Silibinin	75 µM	ATO	2 µM	[47]
Upregulates ATF4 (protein)	T98G	Quercetin	50 µM	Chloroquine	20 µM	[43]
Upregulates CHOP (protein)	T98G	Quercetin	50 µM	Chloroquine	20 µM	[43]
Upregulates Ub (protein)	T98G	Quercetin	50 µM	Chloroquine	20 µM	[43]
Increases [Ca^2+^]_i_	T98G	Quercetin	50 µM	Chloroquine	20 µM	[43]
Increases [Ca^2+^]_m_	T98G	Quercetin	50 µM	Chloroquine	20 µM	[43]
Upregulates ROS	T98G	Quercetin	50 µM	Chloroquine	20 µM	[43]
Decreases autophagy	U87MG	GJ	500 µg/mL	Cisplatin	1 µM	[44]
	U373MG	GJ	500 µg/mL	Cisplatin	1 µM	[44]
	C6	Quercetin	25 µM	NaB	1 mM	[45]
	T98G	Quercetin	25 µM	NaB	1 mM	[45]
Downregulates LC3-II (protein)	C6	Quercetin	25 µM	NaB	1 mM	[45]
	T98G	Quercetin	25 µM	NaB	1 mM	[45]
Upregulates LC3-II (protein)	T98G	Quercetin	50 µM	Chloroquine	20 µM	[43]
Downregulates p62 (protein)	U87MG	GJ	500 µg/mL	Cisplatin	1 µM	[44]
	U373MG	GJ	500 µg/mL	Cisplatin	1 µM	[44]
Upregulates p62 (protein)	T98G	Quercetin	50 µM	Chloroquine	20 µM	[43]
Downregulates Beclin-1 (protein)	C6	Quercetin	25 µM	NaB	1 mM	[45]
	T98G	Quercetin	25 µM	NaB	1 mM	[45]
Downregulates MMP-2 (protein)	U87MG	Silibinin	75 µM	ATO	2 µM	[47]
Downregulates MMP-9 (protein)	U87MG	Silibinin	75 µM	ATO	1, 2 µM	[47]
Decreases metabolic activity	U87MG	Silibinin	75 µM	ATO	1, 2 µM	[47]

## Abbreviations

Akt	serine-threonine kinase Akt
AMPK	5’ Adenosine Monophosphate-activated Protein Kinase
APNG	AlkylPurine-DNA-N Glycosylase
ATF4	Activating Transcription Factor 4
ATO	Arsenic TriOxide
Bax	Bcl-2 associated x protein
BBB	Blood-Brain Barrier
Bcl-2	B-cell lymphoma 2
BZD	BenZoDiazepine
Ca^2+^	calcium ion
[Ca^2+^]_ER_	endoplasmic reticulum calcium ion concentration
[Ca^2+^]_i_	intracellular calcium ion concentration
[Ca^2+^]_m_	mitochondrial calcium ion concentration
CD133	Cluster of Differentiation 133
CHOP	C/EBP HOmologous Protein
EGCG	EpiGalloCatechin-3-Gallate
EMT	Epithelial-Mesenchymal Transition
ER	Endoplasmic Reticulum
ERα	Estrogen Receptor αlpha
ERβ	Estrogen Receptor βeta
FOXO	FOrkhead boX O
GBM	GlioBlastoMa
GJ	Gardenia Jasminoides
GRP78	Glucose Regulated Protein 78
GSC	Glioma Stem Cell
GSLC	Glioma Stem-Like Cell
HIF-1/2	Hypoxia-Inducible Factor 1/2
IP_3_R	IP_3_ Receptor
JNK	c-Jun N-terminal Kinase
LC3	Light Chain 3
MAPK	Mitogen Activated Protein Kinase
MCU	Mitochondrial Calcium Uniporter
MGMT	*O*6-MethyGuanine MethylTransferase
MMP-2	Matrix MetalloProteinase-2
MMP-9	Matrix MetalloProteinase-9
mTOR	mammalian Target Of Rapamycin
NaB	sodium butyrate
NF-κB	Nuclear Factor κappa of B cells
P-gp	P-glycoprotein
PARP	Poly (ADP-Ribose) Polymerase
PI3K	PhosphoInositide 3-Kinase
PWE	Pine needle Water Extract
ROS	Reactive Oxygen Species
TMZ	TeMoZolomide
TSC2	Tuberous SClerosis 2
VEGF	Vascular Endothelial Growth Factor
WNT	Wingless-related iNTegration site

## References

[1] Tamimi A.F., Juweid M., De Vleeschouwer S. (2017). Epidemiology and Outcome of Glioblastoma. Glioblastoma.

[2] Johnson D.R., O’Neill B.P. (2012). Glioblastoma survival in the United States before and during the temozolomide era. J. Neuro-Oncol..

[3] Becker K.P., Yu J. (2012). Status quo--standard-of-care medical and radiation therapy for glioblastoma. Cancer J..

[4] Nishikawa R. (2010). Standard therapy for glioblastoma—A review of where we are. Neurol. Med. Chir..

[5] Lara-Velazquez M., Al-Kharboosh R., Jeanneret S., Vazquez-Ramos C., Mahato D., Tavanaiepour D., Rahmathulla G., Quinones-Hinojosa A. (2017). Advances in Brain Tumor Surgery for Glioblastoma in Adults. Brain Sci..

[6] Noch E.K., Ramakrishna R., Magge R. (2018). Challenges in the Treatment of Glioblastoma: Multisystem Mechanisms of Therapeutic Resistance. World Neurosurg..

[7] Zhai K., Brockmuller A., Kubatka P., Shakibaei M., Busselberg D. (2020). Curcumin’s Beneficial Effects on Neuroblastoma: Mechanisms, Challenges, and Potential Solutions. Biomolecules.

[8] Koklesova L., Liskova A., Samec M., Zhai K., Abotaleb M., Ashrafizadeh M., Brockmueller A., Shakibaei M., Biringer K., Bugos O. (2020). Carotenoids in Cancer Metastasis-Status Quo and Outlook. Biomolecules.

[9] Brockmueller A., Sameri S., Liskova A., Zhai K., Varghese E., Samuel S.M., Büsselberg D., Kubatka P., Shakibaei M. (2021). Resveratrol’s Anti-Cancer Effects through the Modulation of Tumor Glucose Metabolism. Cancers.

[10] Liskova A., Samec M., Koklesova L., Samuel S.M., Zhai K., Al-Ishaq R.K., Abotaleb M., Nosal V., Kajo K., Ashrafizadeh M. (2021). Flavonoids against the SARS-CoV-2 induced inflammatory storm. Biomed. Pharmacother..

[11] Koklesova L., Liskova A., Samec M., Zhai K., Al-Ishaq R.K., Bugos O., Šudomová M., Biringer K., Pec M., Adamkov M. (2021). Protective Effects of Flavonoids Against Mitochondriopathies and Associated Pathologies: Focus on the Predictive Approach and Personalized Prevention. Int. J. Mol. Sci..

[12] Liskova A., Samec M., Koklesova L., Brockmueller A., Zhai K., Abdellatif B., Siddiqui M., Biringer K., Kudela E., Pec M. (2021). Flavonoids as an effective sensitizer for anti-cancer therapy: Insights into multi-faceted mechanisms and applicability towards individualized patient profiles. EPMA J..

[13] Samec M., Liskova A., Koklesova L., Mersakova S., Strnadel J., Kajo K., Pec M., Zhai K., Smejkal K., Mirzaei S. (2021). Flavonoids Targeting HIF-1: Implications on Cancer Metabolism. Cancers.

[14] Zhai K., Siddiqui M., Abdellatif B., Liskova A., Kubatka P., Busselberg D. (2021). Natural Compounds in Glioblastoma Therapy: Preclinical Insights, Mechanistic Pathways, and Outlook. Cancers.

[15] Singh B.N., Shankar S., Srivastava R.K. (2011). Green tea catechin, epigallocatechin-3-gallate (EGCG): Mechanisms, perspectives and clinical applications. Biochem. Pharmacol..

[16] Mani R., Natesan V. (2018). Chrysin: Sources, beneficial pharmacological activities, and molecular mechanism of action. Phytochemistry.

[17] Patel K., Patel D.K. (2017). Medicinal importance, pharmacological activities, and analytical aspects of hispidulin: A concise report. J. Tradit. Complement. Med..

[18] Tay K.C., Tan L.T., Chan C.K., Hong S.L., Chan K.G., Yap W.H., Pusparajah P., Lee L.H., Goh B.H. (2019). Formononetin: A Review of Its Anticancer Potentials and Mechanisms. Front. Pharmacol..

[19] Kelly G.S. (2011). Quercetin. Altern. Med. Rev..

[20] Tan H.L., Chan K.G., Pusparajah P., Saokaew S., Duangjai A., Lee L.H., Goh B.H. (2016). Anti-Cancer Properties of the Naturally Occurring Aphrodisiacs: Icariin and Its Derivatives. Front. Pharmacol..

[21] Ganeshpurkar A., Saluja A.K. (2017). The Pharmacological Potential of Rutin. Saudi Pharm. J..

[22] Deep G., Agarwal R. (2010). Antimetastatic efficacy of silibinin: Molecular mechanisms and therapeutic potential against cancer. Cancer Metastasis Rev..

[23] Roos W.P., Batista L.F., Naumann S.C., Wick W., Weller M., Menck C.F., Kaina B. (2007). Apoptosis in malignant glioma cells triggered by the temozolomide-induced DNA lesion O6-methylguanine. Oncogene.

[24] Hoonjan M., Jadhav V., Bhatt P. (2018). Arsenic trioxide: Insights into its evolution to an anticancer agent. J. Biol. Inorg. Chem..

[25] Kim E.L., Wustenberg R., Rubsam A., Schmitz-Salue C., Warnecke G., Bucker E.M., Pettkus N., Speidel D., Rohde V., Schulz-Schaeffer W. (2010). Chloroquine activates the p53 pathway and induces apoptosis in human glioma cells. Neuro-Oncology.

[26] Park C.-M., Park M.-J., Kwak H.-J., Moon S.-I., Yoo D.-H., Lee H.-C., Park I.-C., Rhee C.H., Hong S.-I. (2006). Induction of p53-mediated apoptosis and recovery of chemosensitivity through p53 transduction in human glioblastoma cells by cisplatin. Int. J. Oncol..

[27] Sawada M., Nakashima S., Banno Y., Yamakawa H., Hayashi K., Takenaka K., Nishimura Y., Sakai N., Nozawa Y. (2000). Ordering of ceramide formation, caspase activation, and Bax/Bcl-2 expression during etoposide-induced apoptosis in C6 glioma cells. Cell Death Differ..

[28] Engelhard H.H., Duncan H.A., Kim S., Criswell P.S., Van Eldik L. (2001). Therapeutic effects of sodium butyrate on glioma cells in vitro and in the rat C6 glioma model. Neurosurgery.

[29] Kanzawa T., Zhang L., Xiao L., Germano I.M., Kondo Y., Kondo S. (2005). Arsenic trioxide induces autophagic cell death in malignant glioma cells by upregulation of mitochondrial cell death protein BNIP3. Oncogene.

[30] Bureta C., Saitoh Y., Tokumoto H., Sasaki H., Maeda S., Nagano S., Komiya S., Taniguchi N., Setoguchi T. (2019). Synergistic effect of arsenic trioxide, vismodegib and temozolomide on glioblastoma. Oncol. Rep..

[31] Sotelo J., Briceno E., Lopez-Gonzalez M.A. (2006). Adding chloroquine to conventional treatment for glioblastoma multiforme: A randomized, double-blind, placebo-controlled trial. Ann. Intern. Med..

[32] Chen T.C., Wang W., Golden E.B., Thomas S., Sivakumar W., Hofman F.M., Louie S.G., Schonthal A.H. (2011). Green tea epigallocatechin gallate enhances therapeutic efficacy of temozolomide in orthotopic mouse glioblastoma models. Cancer Lett..

[33] Zhang P., Sun S., Li N., Ho A.S.W., Kiang K.M.Y., Zhang X., Cheng Y.S., Poon M.W., Lee D., Pu J.K.S. (2017). Rutin increases the cytotoxicity of temozolomide in glioblastoma via autophagy inhibition. J. Neuro-Oncol..

[34] Souza P.O., Bianchi S.E., Figueiro F., Heimfarth L., Moresco K.S., Goncalves R.M., Hoppe J.B., Klein C.P., Salbego C.G., Gelain D.P. (2018). Anticancer activity of flavonoids isolated from Achyrocline satureioides in gliomas cell lines. Toxicol. In Vitro.

[35] Elhag R., Mazzio E.A., Soliman K.F.A. (2015). The Effect of Silibinin in Enhancing Toxicity of Temozolomide and Etoposide in p53 and PTEN-mutated Resistant Glioma Cell Lines. Anticancer Res..

[36] Yang L., Wang Y., Guo H., Guo M. (2015). Synergistic Anti-Cancer Effects of Icariin and Temozolomide in Glioblastoma. Cell Biochem. Biophys..

[37] Wang Y., Liu W., He X., Fei Z. (2015). Hispidulin enhances the anti-tumor effects of temozolomide in glioblastoma by activating AMPK. Cell Biochem. Biophys..

[38] Zhang Y., Wang S.X., Ma J.W., Li H.Y., Ye J.C., Xie S.M., Du B., Zhong X.Y. (2015). EGCG inhibits properties of glioma stem-like cells and synergizes with temozolomide through downregulation of P-glycoprotein inhibition. J. Neuro-Oncol..

[39] Zhang X., Ni Q., Wang Y., Fan H., Li Y. (2018). Synergistic Anticancer Effects of Formononetin and Temozolomide on Glioma C6 Cells. Biol. Pharm. Bull..

[40] Ni Q., Fan Y., Zhang X., Fan H., Li Y. (2019). In vitro and in vivo Study on Glioma Treatment Enhancement by Combining Temozolomide with Calycosin and Formononetin. J. Ethnopharmacol..

[41] Liao C.L., Chen C.M., Chang Y.Z., Liu G.Y., Hung H.C., Hsieh T.Y., Lin C.L. (2014). Pine (*Pinus morrisonicola* Hayata) needle extracts sensitize GBM8901 human glioblastoma cells to temozolomide by downregulating autophagy and O(6)-methylguanine-DNA methyltransferase expression. J. Agric. Food Chem..

[42] Xie C.R., You C.G., Zhang N., Sheng H.S., Zheng X.S. (2018). Epigallocatechin Gallate Preferentially Inhibits O(6)-Methylguanine DNA-Methyltransferase Expression in Glioblastoma Cells Rather than in Nontumor Glial Cells. Nutr. Cancer.

[43] Jang E., Kim I.Y., Kim H., Lee D.M., Seo D.Y., Lee J.A., Choi K.S., Kim E. (2020). Quercetin and chloroquine synergistically kill glioma cells by inducing organelle stress and disrupting Ca^2+^ homeostasis. Biochem. Pharmacol..

[44] Kim H.I., Hong S.H., Ku J.M., Kim M.J., Ju S.W., Chang S.W., Cheon C., Ko S.G. (2020). Gardenia jasminoides Enhances CDDP-Induced Apoptosis of Glioblastoma Cells via AKT/mTOR Pathway While Protecting Death of Astrocytes. Nutrients.

[45] Taylor M.A., Khathayer F., Ray S.K. (2019). Quercetin and Sodium Butyrate Synergistically Increase Apoptosis in Rat C6 and Human T98G Glioblastoma Cells Through Inhibition of Autophagy. Neurochem. Res..

[46] Gulden M., Appel D., Syska M., Uecker S., Wages F., Seibert H. (2017). Chrysin and silibinin sensitize human glioblastoma cells for arsenic trioxide. Food Chem. Toxicol..

[47] Dizaji M.Z., Malehmir M., Ghavamzadeh A., Alimoghaddam K., Ghaffari S.H. (2012). Synergistic effects of arsenic trioxide and silibinin on apoptosis and invasion in human glioblastoma U87MG cell line. Neurochem. Res..

[48] Vargas J.E., Filippi-Chiela E.C., Suhre T., Kipper F.C., Bonatto D., Lenz G. (2014). Inhibition of HDAC increases the senescence induced by natural polyphenols in glioma cells. Biochem. Cell Biol..

[49] Chou T.C. (2010). Drug combination studies and their synergy quantification using the Chou-Talalay method. Cancer Res..

[50] Hu J., Webster D., Cao J., Shao A. (2018). The safety of green tea and green tea extract consumption in adults—Results of a systematic review. Regul. Toxicol. Pharmacol..

[51] Lu N.T., Crespi C.M., Liu N.M., Vu J.Q., Ahmadieh Y., Wu S., Lin S., McClune A., Durazo F., Saab S. (2016). A Phase I Dose Escalation Study Demonstrates Quercetin Safety and Explores Potential for Bioflavonoid Antivirals in Patients with Chronic Hepatitis C. Phytother. Res..

[52] Sharma S., Ali A., Ali J., Sahni J.K., Baboota S. (2013). Rutin: Therapeutic potential and recent advances in drug delivery. Expert Opin. Investig. Drugs.

[53] Barcena R., Moreno A., Rodriguez-Gandia M.A., Albillos A., Arocena C., Blesa C., Garcia-Hoz F., Graus J., Nuno J., Lopez-Hervas P. (2013). Safety and anti-HCV effect of prolonged intravenous silibinin in HCV genotype 1 subjects in the immediate liver transplant period. J. Hepatol..

[54] Brown E.S., Bice C., Putnam W.C., Leff R., Kulikova A., Nakamura A., Ivleva E.I., Enkevort E.V., Holmes T., Miingi N. (2019). Human Safety and Pharmacokinetics Study of Orally Administered Icariin: Randomized, Double-Blind, Placebo-Controlled Trial. Nat. Prod. Commun..

[55] Ong S.K.L., Shanmugam M.K., Fan L., Fraser S.E., Arfuso F., Ahn K.S., Sethi G., Bishayee A. (2019). Focus on Formononetin: Anticancer Potential and Molecular Targets. Cancers.

[56] Liu K., Zhao F., Yan J., Xia Z., Jiang D., Ma P. (2020). Hispidulin: A promising flavonoid with diverse anti-cancer properties. Life Sci..

[57] Kavvadias D., Sand P., Youdim K.A., Qaiser M.Z., Rice-Evans C., Baur R., Sigel E., Rausch W.D., Riederer P., Schreier P. (2004). The flavone hispidulin, a benzodiazepine receptor ligand with positive allosteric properties, traverses the blood-brain barrier and exhibits anticonvulsive effects. Br. J. Pharmacol..

[58] El-Bakoush A., Olajide O.A. (2018). Formononetin inhibits neuroinflammation and increases estrogen receptor beta (ERbeta) protein expression in BV2 microglia. Int. Immunopharmacol..

[59] Li S., Dang Y., Zhou X., Huang B., Huang X., Zhang Z., Kwan Y.W., Chan S.W., Leung G.P., Lee S.M. (2015). Formononetin promotes angiogenesis through the estrogen receptor alpha-enhanced ROCK pathway. Sci. Rep..

[60] Mereles D., Hunstein W. (2011). Epigallocatechin-3-gallate (EGCG) for clinical trials: More pitfalls than promises?. Int. J. Mol. Sci..

[61] Angeloni C., Barbalace M.C., Hrelia S. (2019). Icariin and Its Metabolites as Potential Protective Phytochemicals against Alzheimer’s Disease. Front. Pharmacol..

[62] Liskova A., Koklesova L., Samec M., Smejkal K., Samuel S.M., Varghese E., Abotaleb M., Biringer K., Kudela E., Danko J. (2020). Flavonoids in Cancer Metastasis. Cancers.

[63] Cassidy A., Minihane A.M. (2017). The role of metabolism (and the microbiome) in defining the clinical efficacy of dietary flavonoids. Am. J. Clin. Nutr..

[64] Guo Y., Bruno R.S. (2015). Endogenous and exogenous mediators of quercetin bioavailability. J. Nutr. Biochem..

[65] Pervin M., Unno K., Nakagawa A., Takahashi Y., Iguchi K., Yamamoto H., Hoshino M., Hara A., Takagaki A., Nanjo F. (2017). Blood brain barrier permeability of (-)-epigallocatechin gallate, its proliferation-enhancing activity of human neuroblastoma SH-SY5Y cells, and its preventive effect on age-related cognitive dysfunction in mice. Biochem. Biophys. Rep..

[66] Wang M., Rong Y., Luo L. (2021). Neuroprotective effects of icariin in neonatal hypoxia-ischemic brain damage via its anti-apoptotic property. Childs Nerv. Syst..

[67] da Silva A.B., Cerqueira Coelho P.L., das Neves Oliveira M., Oliveira J.L., Oliveira Amparo J.A., da Silva K.C., Soares J.R.P., Pitanga B.P.S., Dos Santos Souza C., de Faria Lopes G.P. (2020). The flavonoid rutin and its aglycone quercetin modulate the microglia inflammatory profile improving antiglioma activity. Brain Behav. Immun..

[68] Speciale A., Muscara C., Molonia M.S., Cimino F., Saija A., Giofre S.V. (2021). Silibinin as potential tool against SARS-Cov-2: In silico spike receptor-binding domain and main protease molecular docking analysis, and in vitro endothelial protective effects. Phytother. Res..

[69] Lee S.Y. (2016). Temozolomide resistance in glioblastoma multiforme. Genes Dis..

[70] Ahmed E.M., Bandopadhyay G., Coyle B., Grabowska A. (2018). A HIF-independent, CD133-mediated mechanism of cisplatin resistance in glioblastoma cells. Cell. Oncol..

[71] Taki T., Ohnishi T., Arita N., Hiraga S., Hayakawa T. (1998). In vivo etoposide-resistant C6 glioma cell line: Significance of altered DNA topoisomerase II activity in multi-drug resistance. J. Neuro-Oncol..

